# Stochasticity, Bistability and the Wisdom of Crowds: A Model for Associative Learning in Genetic Regulatory Networks

**DOI:** 10.1371/journal.pcbi.1003179

**Published:** 2013-08-22

**Authors:** Matan Sorek, Nathalie Q. Balaban, Yonatan Loewenstein

**Affiliations:** 1 Edmond and Lily Safra Center for Brain Sciences and the Interdisciplinary Center for Neural Computation, The Hebrew University of Jerusalem, Jerusalem, Israel; 2 Department of Genetics, The Alexander Silberman Institute of Life Sciences, The Hebrew University of Jerusalem, Jerusalem, Israel; 3 Racah Institute of Physics, Center for Nanoscience and Nanotechnology and Sudarsky Center for Computational Biology, The Hebrew University of Jerusalem, Jerusalem, Israel; 4 Department of Neurobiology and the Center for the Study of Rationality, The Hebrew University of Jerusalem, Jerusalem, Israel; Cajal Institute, Consejo Superior de Investigaciones Científicas, Spain

## Abstract

It is generally believed that associative memory in the brain depends on multistable synaptic dynamics, which enable the synapses to maintain their value for extended periods of time. However, multistable dynamics are not restricted to synapses. In particular, the dynamics of some genetic regulatory networks are multistable, raising the possibility that even single cells, in the absence of a nervous system, are capable of learning associations. Here we study a standard genetic regulatory network model with bistable elements and stochastic dynamics. We demonstrate that such a genetic regulatory network model is capable of learning multiple, general, overlapping associations. The capacity of the network, defined as the number of associations that can be simultaneously stored and retrieved, is proportional to the square root of the number of bistable elements in the genetic regulatory network. Moreover, we compute the capacity of a clonal population of cells, such as in a colony of bacteria or a tissue, to store associations. We show that even if the cells do not interact, the capacity of the population to store associations substantially exceeds that of a single cell and is proportional to the number of bistable elements. Thus, we show that even single cells are endowed with the computational power to learn associations, a power that is substantially enhanced when these cells form a population.

## Author Summary

It has been known since the pioneering studies of Ivan Petrovich Pavlov that changes in the nervous system enable animals to associate neutral stimuli with stimuli of ecological significance. The goal of this paper is to study whether genetic regulatory networks that govern the production and degradation of proteins in all living cells are capable of a similar associative learning. We show that a standard model of a genetic regulatory network is capable of learning multiple overlapping associations, similar to a neural network. These results demonstrate that even bacteria that are devoid of a nervous system can learn associations. Moreover, as cells often reside in large clonal populations, as in a colony of bacteria or in tissue, we consider the ability of a large population of identical cells to learn associations. We show that even if the cells do not interact, the computational capabilities of the population far exceed those of the single cell. This result is a first demonstration of “wisdom of crowds” in clonal populations of cells. Finally, we provide specific guidelines for the experimental detection of associative learning in populations of bacteria, a phenomenon that may have been overlooked in standard experiments.

## Introduction

### Associative learning

**Figure 1:**
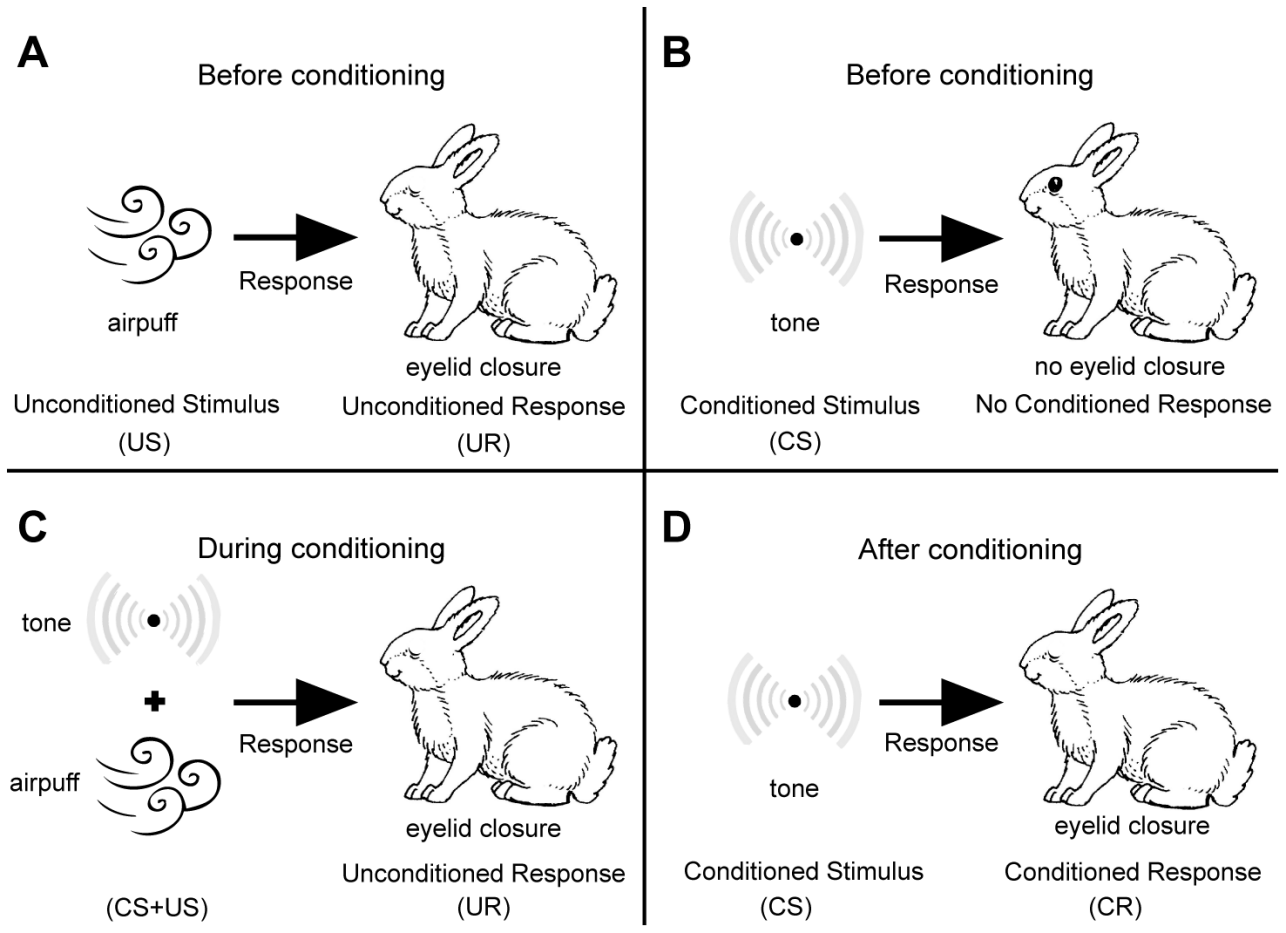
A schematic illustration of eye-blink conditioning. (A) Naïve animal responds to the presentation of an airpuff (the US) by eyelid closure. (B) By contrast, a tone (the CS) does not elicit any overt response. (C) During conditioning the CS and the US are repeatedly paired. (D) After conditioning the animal responds to the CS with eyelid closure (the CR).

Almost all animals can associate neutral stimuli and stimuli of ecological significance [1]. An extensively studied example is eye-blink conditioning (Figure 1) [2], [3]. Naïve rabbits respond to an airpuff to the cornea (Unconditioned Stimulus, US) with eyelid closure (Unconditioned Response, UR). By contrast, a weak auditory or visual stimulus (Conditioned Stimulus, CS) does not elicit such an overt response. Repeated pairing of the CS and the US forms a cognitive association between the CS and the US such that the trained animal responds to the CS with eyelid closure, a response known as Conditioned Response (CR). Two important characteristics of associative learning are (1) specificity and (2) generality. The CR does not reflect a general arousal. Rather, the animal learns to respond specifically to the CS. The generality is reflected by the fact that a large family of potential stimuli can serve as a CS if paired with the US.

Neuronal networks are particularly adapted to performing this association and in the last few decades there has been considerable progress in understanding the ways in which experience-based changes in synapses in the nervous system underlie this associative learning process [4], [5]. Neural network models for associative memory, which explain how both specificity and generality are maintained, are typically based on three elements: (1) Synapses are the physical loci of the memory; (2) synaptic plasticity underlies memory encoding; (3) neural network dynamics, in which the activities of neurons depend on the synaptic efficacies, underlie the retrieval of the learned memories in response to the CS.

### Genetic regulatory networks

Genetic regulatory networks (GRN) describe the interaction of genes in the cell through their RNA and protein products [6], [7], [8]. Previous studies have pointed out the similarity between the dynamics of GRNs and the dynamics of neural networks [9]. For example, GRNs, like neural networks, can implement logic-like circuits, where the concentration of a protein (high or low) corresponds to the binary state of the gate [10], [11], [12]. These findings prompted us to evaluate the capacity of GRNs to learn associations.

Considering associative learning in animals, the US is typically a stimulus of biological significance, such as food or a noxious stimulus that elicits a response (UR) in the naïve animal, either in the form of muscle activation or gland secretion. The GRN correlate of a pain-inducing stimulus is stress. Stressful conditions such as heat, extreme pH, or toxic chemicals often result in a substantial change in the expression level of many different proteins in the cell. For example, *Escherichia coli* (*E. coli*) bacteria respond to a variety of stress conditions by a general stress response mechanism in which the master regulator 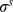 controls the expression of many genes [13]. These stressful conditions can be regarded as a US and the resultant change in the expression level of the proteins can be regarded as a UR. By contrast, other stimuli may result in a narrow or absence of a response of the cell and in that sense can be referred to as potential CS. Learning in this framework would correspond to the formation of an association between these potential CS and US such that following the repeated pairing of the CS and US, the presentation of the CS would elicit a UR-like response (CR).

The responsiveness of the GRNs to different stimuli has been shown to change over time in response to evolutionary pressure in a manner that resembles associative learning [14], [15]. These changes take place on time scales that are substantially longer than the lifetime of a single cell and in contrast to associative learning in animals, entail modifications of the genome through mutations. On a shorter timescale, there is some evidence that the single-celled *Paramecium* can learn to associate a CS with a US within its lifetime [16]. However, these findings have been disputed [17] and the question of whether *Paramecia* can learn associations and the characteristics of this learning await further experimental validation. The capacity of GRNs to learn associations in shorter, non-evolutionary time-scales has also been studied theoretically using GRN models. Learning in these models is restricted to a small subset of predefined stimuli [18], [19], [20], [21] and thus the computational capabilities of these GRN models are limited compared to neural network models.

Here we show that a GRN based on bistable elements and stochastic transitions can learn associations while retaining both specificity and generality. We further compute the capacity of the network and show that the number of different learned associations that the network can simultaneously retain is proportional to the square root of the number of bistable elements. Moreover, this capacity is substantially enhanced when considering a clonal population of GRNs. These results imply that even bacteria are endowed with the capacity to learn multiple associations.

## Results

Our Genetic Associative Memory model (GAM) for associative learning is based on three components: (1) a memory module that provides the long time-scale necessary for the maintenance of memories for long periods of time; (2) a mechanism for encoding the desired memories and (3) a response mechanism for the readout or retrieval of the stored memories in response to the relevant stimuli. We describe the three components separately in the simpler case of a predefined association and then generalize to the case of multiple associations and to the case of a population of GAMs.

### Learning a predefined association

#### Memory

A necessary condition for associative learning is the ability to maintain memories. Memories require a long time-scale, which characterizes multistable dynamics [22], [23]. For example, the ability of flip-flop devices in electronic circuits to maintain memories is based on their bistable dynamics. Bistability naturally emerges in dynamical systems if two conditions are fulfilled: positive feedback and saturation [24], [25], [26]. Both these requirements characterize many GRNs, and bistability has been found in both artificially engineered [27], [28], [29], [30] and natural GRNs [31], [32], [33], [34], [35], [36]. For example, the response of the lactose promoter in *E. coli* to intermediate induction levels was shown to be bistable because of the positive feedback loop on the import of the inducer in the cell [37].

In our GAM, we assume a positive feedback loop between a gene and its protein product. The gene encodes for a protein *M* which binds cooperatively, as a transcription factor, to the promoter of that gene, resulting in further synthesis of *M*. The kinetic reactions describing the dynamics of *M* appear in Text S1 in the Supporting Information, and their deterministic approximation [7], [8] is equivalent to those of positive feedback loops such as the lac system [38], [39]:
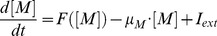
(1)where 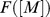 reflects the nonlinear positive feedback (see Eq. (4) in the Materials and Methods). The second term in Eq. (1) denotes the protein degradation, where 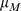 is a parameter. The third term models the contribution of external factors to the dynamics of *M* (see below).

**Figure 2:**
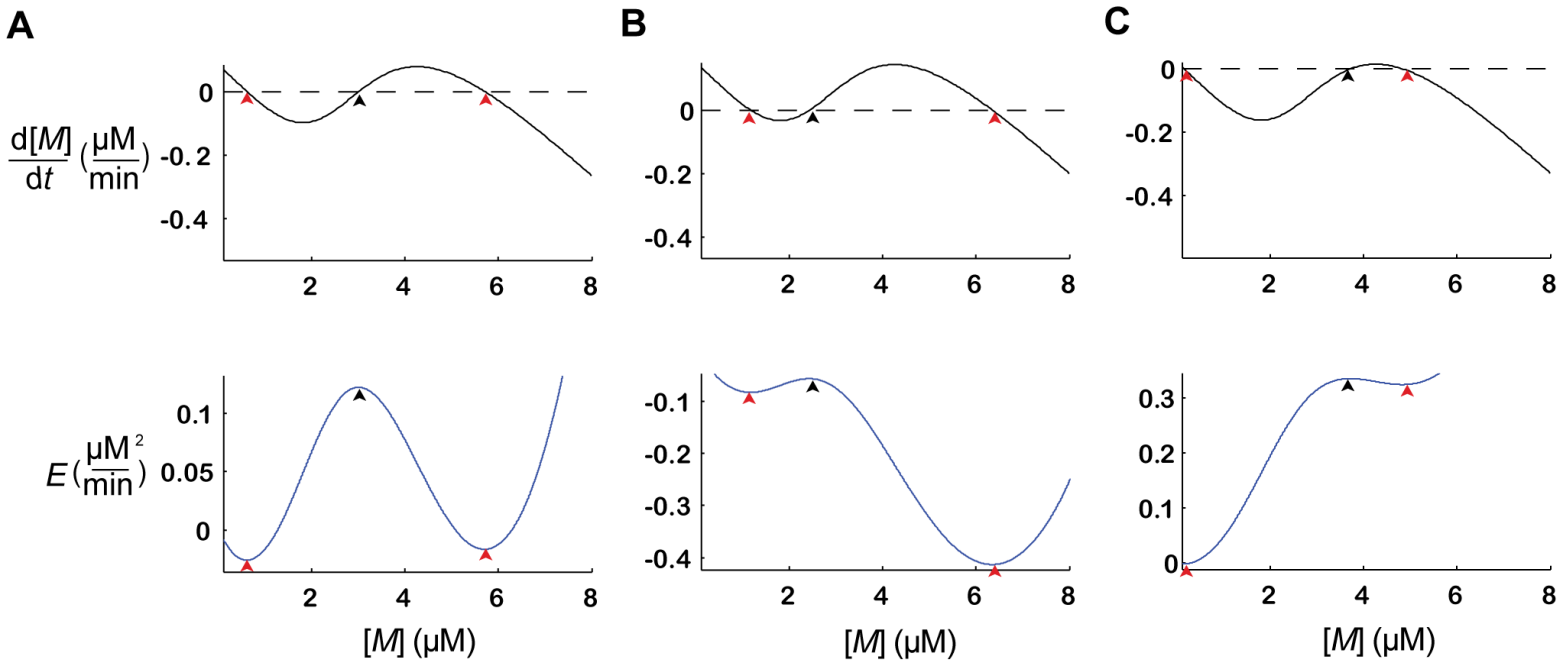
The bistable dynamics of the memory element. (**A–C**) The dynamics described by Eq. (1) for three different values of 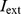. Top, 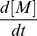; bottom, the corresponding energy function 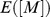. The red and black arrows denote the stable and unstable fixed points, respectively (zero crossings of 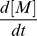, top and extrema of the energy function, bottom). The value of 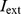 determines the offset of 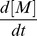 and hence the energy gaps. (**B**) The larger 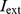 is, the smaller the energy gap corresponding to 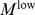. (**C**) The smaller 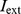 is, the smaller the energy gap corresponding to 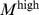. The values of the external inputs are 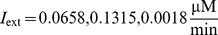 in A–C, respectively.

The function 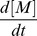, depicted in Figure 2A (top), is N-shaped and is characterized by three zero-crossings. The two outermost zero-crossings (red arrows in Figure 2A, top) correspond to the two stable states (or fixed-points): a low expression level of *M*, 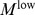 (left) and a high expression level, 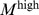 (right). It can be readily shown that the intermediate zero-crossing (black arrow) corresponds to an unstable fixed-point of the dynamics. Thus, the dynamics of Eq. (1) converge to one of the equilibrium values, the low or high expression level of *M*, depending on the initial conditions. This bistability of the dynamics of *M* endows the GRN with the capacity to store binary memories in the form of the level of expression of *M*. For this reason we refer to *M* as a ‘pseudo-synapse’.

It is useful to rewrite Eq. (1) using an ‘energy’ function such that 
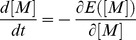
 where 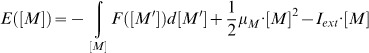. The energy function 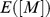 (Figure 2A, bottom) is characterized by two minima (red arrows) and one local maximum (black arrow). The two minima correspond to the two stable fixed points of the dynamics of *M* and the maximum corresponds to the intermediate unstable fixed point.

The differences between the value of the energy function at the maximum and the values at the minima are known as the energy gaps and are denoted by 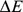. In Figure 2A the two energy gaps are approximately equal. However, an increase in the value of 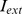 raises the function 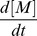 (Figure 2B top), resulting in a smaller energy gap for the 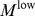 fixed point, and a larger energy gap for 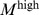 fixed point (Figure 2B bottom). By contrast, a decrease in the value of 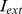 lowers the function 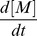 (Figure 2C top), resulting in the opposite effect: a larger energy gap for 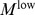, and a smaller energy gap for 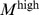 (Figure 2C bottom).

It should be noted that Eq. (1) is a deterministic approximation of the biochemical dynamics. Biochemical processes such as the bursting activity of the transcriptional machinery are stochastic [40], [41], [42], [43]. One way to account for the stochasticity is by adding white noise to Eq. (1) such that
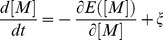
(2)where 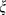 is a Gaussian white noise, 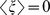, 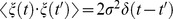, 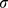 is the magnitude of the noise and 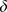 is the Dirac delta “function”.

One consequence of this stochasticity is that the noise is expected to occasionally induce transitions between the two fixed points. A well-known result from the field of stochastic processes is that if the noise is sufficiently weak, the rate of transitions 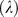 from one minimum to the other is exponentially dependent on the energy gap, 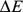, 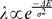 (e.g., see [44], and see also [45] for a more accurate approximation). Consequently, even a small change in the energy gap is expected to result in a large change in the transition rate. Thus, although the three energy functions in Figure 1A–C are qualitatively similar, they represent very different dynamics. For sufficiently weak noise, the rate of transition from 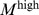 to 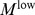 in Figure 2B is negligible compared to the rate of transition from 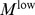 to 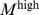. Similarly, the rate of transition from 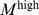 to 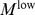 in Figure 2C is negligible compared to the rate of transition from 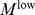 to 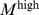. Moreover, the rates of transition between the two stable states in Figure 2A are both negligible compared to the rate of transition from 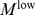 to 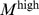 in Figure 2B or the rate of transition from 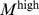 to 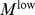 in Figure 2C. Thus, the transitions between the states are highly dependent on the value of 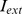. We utilize these results when modeling the memory encoding in the next section.

#### Encoding

In associative learning, memory is encoded in response to the contiguity of the CS and the US. To implement this idea in the framework of the GAM, we assume that the value of 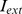 is determined by external cues, the CS and US. Formally, we assume that the CS and the US induce the expression of proteins *C* and *U*, respectively. The value of 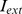 is determined by the concentrations of *C* and *U* such that the US is effectively a repressor of *M* but the co-occurrence of US and CS activates the expression of *M* (see Eq. (4) in the Materials and Methods). In other words, *U* in isolation decreases 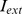 but when bound to *C* it increases 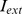. This mode of regulation has already been observed; e.g., in the osmotic response regulatory system in yeast [46].

For simplicity we assume in our model that the expression levels of *C* and *U* are binary, 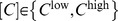 and 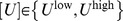, reflecting the presence or absence of the CS and US, respectively. Moreover, we assume that independently of the external cues, the value of 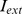 is such that the dynamics of the pseudo-synapse are bistable (as in Figure 2). Thus, the co-occurrence of the CS and US increases the transition rate to the high expression level of *M* (as in Figure 2B) whereas an exposure of the GAM to the US alone increases the transition rate to the low expression level of *M* (as in Figure 2C).

The computational implications of these dynamics are that a repeated exposure of the GAM to the co-occurrence of the CS and US is expected to result in a high state of *M*, whereas a repeated exposure of the GAM to the US in the absence of the CS is expected to result in a low state of *M*. In this sense, the state of *M* is the physical correlate of the memory of the association between the CS and US and a high level of *M* indicates an association between the CS and US. Assuming that in the absence of the US, the two energy gaps are high (as in Figure 2A), the transition rates between the two states of the pseudo-synapse, in both directions, would be low. Thus, in the absence of the US, information about the existence of an association, as well as its absence, would be maintained for long periods of time. These dynamics are reminiscent of a multiplexer. A multiplexer is a device that selects one of several input signals and forwards the selected input into a single line. In the dynamics of *M*, the US selects whether *M* will be maintained (in the absence of the US) or whether the value of *M* is determined by the CS (in the presence of the US), as in [18]. However, in contrast to a standard multiplexer, transitions in our model are stochastic. Thus, the dynamics depicted in Figure 2 resemble a stochastic multiplexer. This difference implies that multiple repetitions are needed in order to change the state of *M* with a high probability.

#### Retrieval

**Figure 3:**
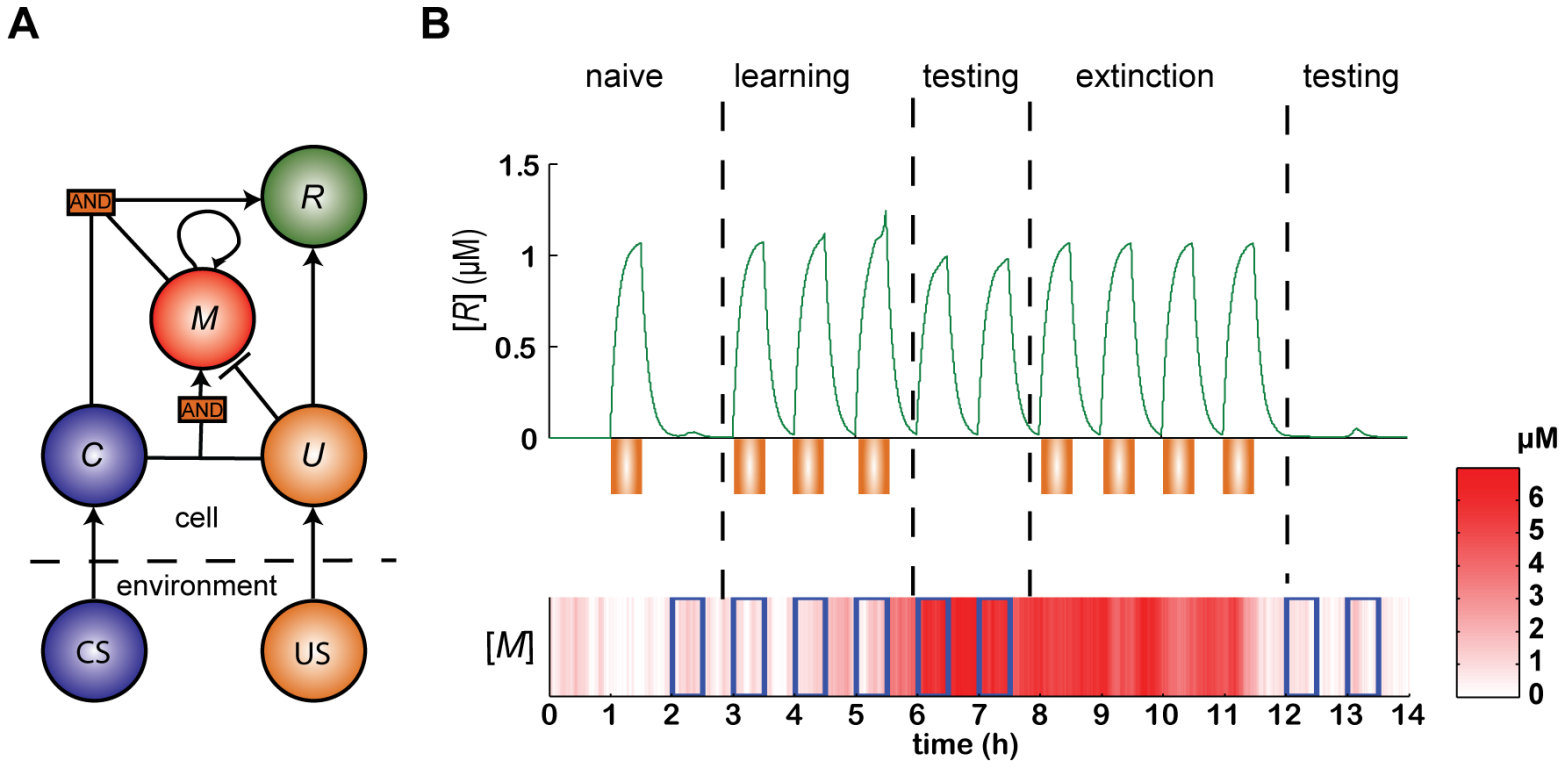
A model for a Genetic Associative Memory module (GAM). (A) A logic circuit representing the GRN's regulatory dynamics. The external signals CS (blue) and US (orange) induce the expression of the proteins *C* (blue) and *U* (orange), respectively. The expression of *U* elicits a response *R* (green) independently of *C*. In contrast, *C* elicits a response *R* only if the expression level of *M* (red) is high. The expression of *M* is induced by a high concentration of *M* (the positive feedback, Eq. (2)) or by the co-expression of *C* and *U*, and is inhibited by the expression of *U* in the absence of *C*. (B) Associative learning in a simulation of the GAM. Initially, the GAM is in the naïve state, in which 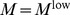. In this state the GAM responds to the US (orange) but not to the CS (blue rectangles). Repeated pairing of the CS and US (*t* = 3, 4 and 5 h) changes the state of *M* (color coded in brightness) to the high state (immediately after *t* = 5 h). As a result, the GAM is responsive to the CS in isolation (*t* = 6 and 7 h). In response to repeated presentation of the US in the absence of the CS (*t* = 8, 9, 10 and 11 h), the expression level of *M* reverts to the low state (immediately after *t* = 11 h) resulting in a loss of response to the learned CS (*t* = 12 and 13 h). Note that the response at *t* = 5 h is slightly higher than the responses at previous times. This results from the transition of *M* to its high state.

The last component of our GAM is a readout scheme that decodes the state of *M* in the presence of a CS such that the CS elicits a response if and only if the expression level of *M* is high. To implement this, we assume that the UR and the CR manifest in the GAM as the production of a response protein *R*. The expression of *R* is regulated by two mechanisms: the US regulates the expression of *R* through the binding of *U* to a promoter of *R*, and the CS-pseudo-synapse pair regulates the expression of *R* by cooperative binding of *C* and *M* to another promoter (Figure 3A). The kinetic reactions describing the dynamics of the expression of *R* appear in Text S1 in the Supporting Information, and their deterministic approximation is given by:
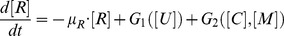
(3)where 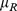 is the degradation rate of *R* and the functions 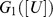 and 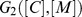 describe the dependence of the expression of *R* on *U*, *C* and *M* (see Eq. (5) in the Materials and Methods). A high level of *U* in Eq. (3) results in a high value of 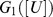. This elicits the expression of *R*, independently of the values of *M* and *C*, corresponding to the UR. By contrast, a high level of *C* results in a high level of 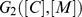 only when *M* is in its high expression level. Thus, in the absence of the US, the stimulus substantially increases *R* only when *M* is in its high expression level, corresponding to the CR.

The dynamics of Eqs. (1–3) describe a GAM that can learn the association between a CS and a US. This is demonstrated in Figure 3B. Initially, at time *t* = 0, *M* is in the low expression level state, corresponding to the ‘naïve’ state of the network prior to learning. In this state, a US (orange rectangle, *t* = 1 h) elicits a response (UR), but a CS (open blue rectangle, *t* = 2 h) does not elicit any response. Following two pairings of the CS and US (*t* = 3 and 4 h), the state of *M* does not change but in response to the third pairing (*t* = 5 h) the state of *M* changes to the high state level. In this state, the GAM is responsive to both a CS (*t* = 6 and 7 h) and a US (*t* = 8 h). Three presentations of the US in the absence of a CS (*t* = 8–10 h) do not elicit any change in the state of *M* but in response to another presentation of the US in the absence of a CS (*t* = 11 h), the state of *M* reverts to its low value, and the GAM is no longer responsive to CS (at *t* = 12 and 13 h).

### Learning multiple associations

In the previous section, we demonstrated that a GRN can learn to associate a CS with a US (Figure 3). However, this learning is limited, as it is specific to a single, predefined CS. This GAM can be trivially generalized to enable the learning of several different associations by postulating that the GAM is characterized by a number of memory elements, each associated with a single CS. However, this generalized GAM is still limited in its ability to learn associations because only those predefined CS can be learned. This limitation contrasts sharply with neural network models, which are capable of learning general associations. In this section we generalize the model presented in Figure 3A and show that similar to neural network models, GRNs are also endowed with the capacity to learn a large number of arbitrary, overlapping CS.

**Figure 4:**
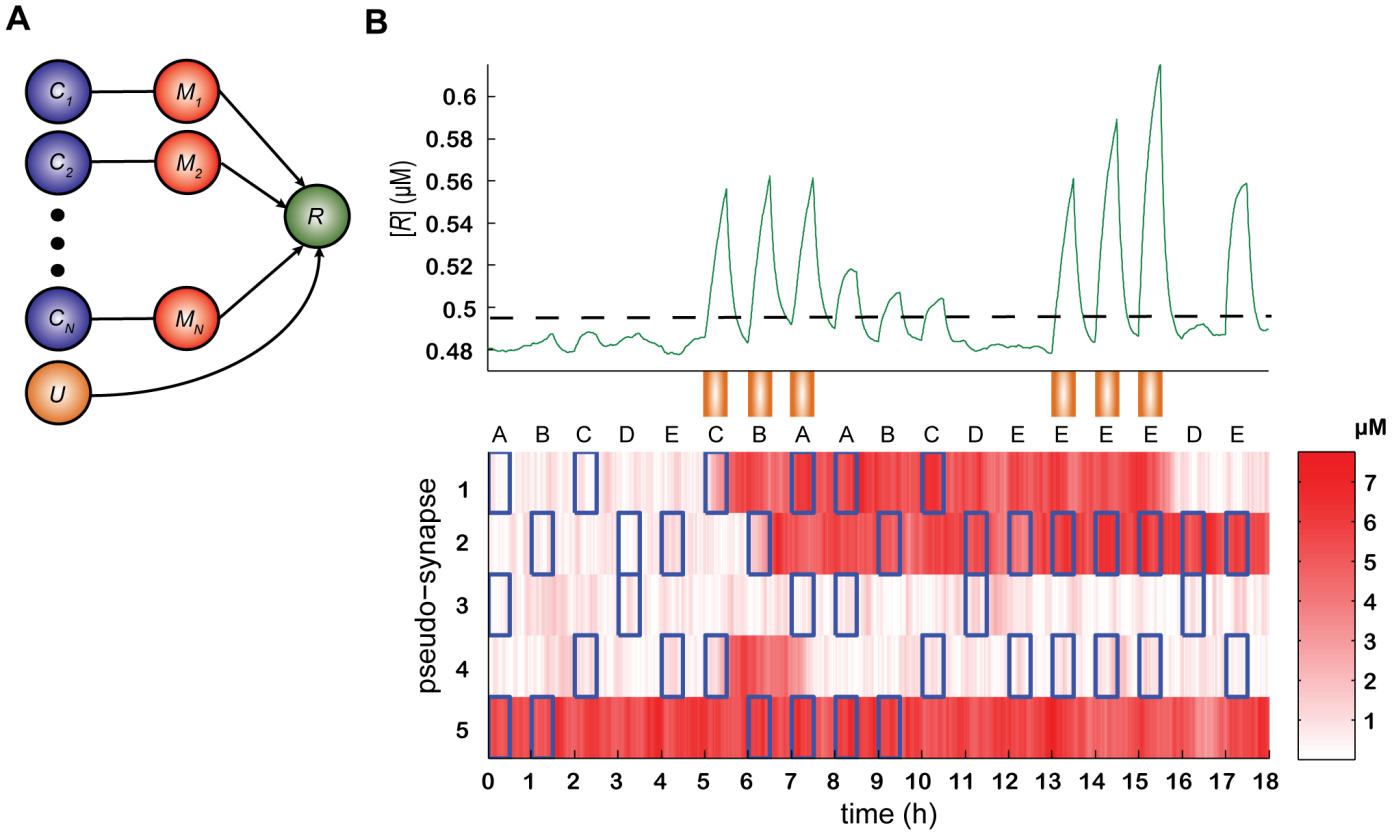
A model for learning multiple overlapping associations. (A) A schematic description of the dependence of the expression of *R* (green) on the activation of the receptors 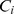 (blue), the pseudo-synapses 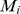 (red) and the US (orange), see Eq. (5). Note that for reasons of clarity, the encoding process, which follows the same dynamics as in Eq. (4) (see Figure 3A) is not shown. (B) Simulation of the model (Eqs. (4) and (5)). Bottom, the expression level of 5 representative pseudo-synapses over time is depicted using a color code (color coded in brightness); green, the response *R*; orange rectangles, the timing of a US; open blue rectangles, the timing of activations of 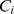 by a stimulus. Initially, the GAM is in a naïve state. In that state, its response to CS (*t* = 0, 1, 2, 3 and 4 h) is below some threshold (dashed horizontal line). In response to the pairing of three of the CS (C, B and A) with the US (*t* = 5, 6 and 7 h, respectively), a fraction of the pseudo-synapses which correspond to an active 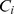 undergo a transition to the high expression state (e.g., *i* = 2 at *t* = 6 h) and a fraction of the pseudo-synapses which correspond to an inactive 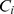 undergo a transition to the low expression state (e.g., *i* = 4 at *t* = 7 h). As a result, the response of the GAM to A, B and C (*t* = 8, 9 and 10 h) is larger than the response to the unlearned stimuli, D and E (*t* = 11 and 12 h, respectively). As a result of repeated association of pattern E with the US (at times *t* = 13, 14 and 15 h), the GAM vigorously responds to the presentation of pattern E (at time *t* = 17 h) but not to pattern D (at time *t* = 16 h).

Consider the network described Figure 4A. In contrast to the single-pathway model (Figure 3A), in which a CS induces the expression of a single protein *C*, in the generalized model we assume that the CS are complex stimuli that activate *N* different receptors, 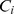, 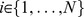. Each receptor 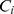 is associated with a single pseudo-synapse 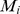. The dynamics of each of the pseudo-synapses follow the same equations as in the single-pathway model (not shown in Figure 4A, see Eq. (4) in the Materials and Methods).

The last component of our generalized GAM is the readout scheme. We assume that similar to the single-pathway model, the UR and the CR manifest in the generalized model as the production of a response protein *R*. We assume two independent promoters that regulate the expression of *R*. The response to the presentation of the US is described by 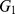 (Eq. (3)) and the response to the presentation of the CS is regulated by the cooperative binding of 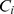 and 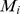, where different pairs of 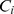 and 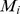 independently regulate *R* (Eq. (5) in the Materials and Methods and Text S1 in Supplementary Information).

For simplicity, we assume in our analysis that the patterns of expression of the proteins 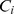 that define the stimuli are random and independent. In this case, the statistics of the stimuli are fully determined by the sparseness of the stimuli, the probability that 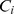 is in its high expression level, 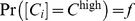.

To gain insights into the ability of the generalized GAM to learn multiple associations, we consider a naïve GAM, in which the values of the pseudo-synapses are random (Figure 4B, bottom, *t* = 0). The responses of the GAM to five different stimuli, denoted by A, B, C, D and E, presented to the GAM at times *t* = 0, 1, 2, 3 and 4 h, respectively, are relatively small. This is due to the random, and hence relatively small overlap between the pattern of activation of pseudo-synapses (color coded) and the pattern of activation of the receptors 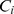 of the five stimuli (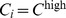 is denoted by an open blue rectangle in Figure 4B).

In response to the pairing of C, B and A with the US (at times *t* = 5, 6 and 7 h, respectively), the expression levels of some of the 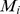 become more similar to that of the 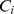 in A, B and C, respectively. As a result, the GAM responds more vigorously to the presentation of A, B and C (at times *t* = 8, 9 and 10 h, respectively) but not to the presentation of D or E (at times *t* = 11 and 12 h, respectively). However, as a result of a repeated association of pattern E with the US (at times *t* = 13, 14 and 15 h), the GAM vigorously responds to the presentation of pattern E (at time *t* = 17 h) but not to pattern D (at time *t* = 16 h). This example demonstrates that a GAM can selectively learn to associate several arbitrary CS patterns with a US.

### Order effect

A careful analysis of Figure 4B reveals that after learning, the magnitude of the responses to the three learned CS is not equal. The response to stimulus C (*t* = 10 h) is smaller than the response to stimulus B (*t* = 9 h) and the response to B is smaller than the response to A (*t* = 8 h). This difference reflects the fact that the order of association affects the magnitude of the response to a CS. This is because learning a new pattern may change the expression level of a pseudo-synapse that participates in the encoding of an older pattern. For example, consider pseudo-synapse 4 in Figure 4B. In response to the presentation of stimulus C (at time *t* = 5 h), the state of the pseudo-synapse has changed to the high expression level, in line with the expression level of 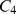 in CS C. However, the association of the US with A (at time *t* = 7 h) has reverted the state of the pseudo-synapse to the low expression level, decreasing the overall response to the CS C. In other words, the association with the CS A has overwritten the information stored in pseudo-synapse 4 concerning the CS C. More generally, because of the overwriting of memories by more recent memories, the magnitude of response to a CS is expected to decrease with the number of subsequent CSs. After the encoding of a large number of patterns, the response to an ‘old’ CS is expected to diminish to an extent where it is no longer distinguishable from the response to non-learned stimuli. In this case the CS is said to have been extinguished (a more precise definition of “distinguishable” appears below). By contrast to the diminishing of the response to a pattern following the overwriting by other patterns, the repeated co-occurrence of the same pattern with the US (at times *t* = 13, 14 and 15 h) augments the strength of association of that pattern with the US, as demonstrated by the response to pattern E at time *t* = 17 h.

The magnitude of the order effect depends on two probabilities: the probability *p* that the co-occurrence of *U* and a high level of expression of 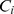 would induce a transition from 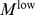 to 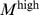 in the corresponding pseudo-synapse 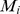 and the probability *q* that the co-occurrence of *U* and a low level of expression of the corresponding 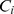 would induce a transition from 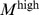 to 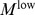 in the corresponding pseudo-synapse 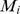. The probabilities *p* and *q* are determined by the two rates of the US-induced transitions and the duration of co-occurrence of the US and CS, *T* (assuming that the rates of all other transitions are negligible, see above) such that 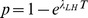 and 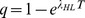, where 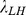 and 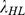 are the low-to-high and high-to-low transition rates, respectively. The larger the transition rates and the longer the duration, the larger the transition probabilities are.

If 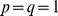 all pseudo-synapses are determined by the most recent CS and the pattern of expression level of the different pseudo-synapses corresponds to the pattern of activation of the receptors in that CS. As a result, the response to the most recent CS is substantially larger than the response to a non-learned stimulus. However, this comes at a price. The most recent CS overwrites the memory trace of all previously encoded CS and therefore the responses to all these ‘older’ CS are indistinguishable from the responses to the non-learned stimuli. Thus, if 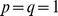, the GAM cannot store more than a single association. The smaller the values of *p* and *q* (e.g., due to smaller US-induced transition rates), the fewer pseudo-synapses change in the process of learning a CS, allowing the GAM to maintain information about previously-learned CSs.

However, the transition probabilities should not be too small because the smaller these probabilities are, the weaker is the encoding. If these probabilities are too small, the response of the GAM even to the most recently stored GAM is too small to be distinguishable from non-learned stimuli. Therefore, in order for the GAM to be able store a large number of CS, the values of the US-induced transition rates should be sufficiently large to allow for a sufficiently large response to the learned-CS but sufficiently small to minimize the overwriting of old memories by new memories.

To better understand the requirement that the response to a CS needs to be distinguishable from the response to non-learned stimuli, consider again Figure 4B. The responses of the GAM to the presentations of the non-learned stimuli A-E at times 0–5 h, respectively, are not identical. These differences are due to the fact that there is stochasticity in the response, resulting from stochasticity in the dynamics of the pseudo-synapses and in the realization of the different CS. Therefore, a memory of a CS is said to be maintained if the *distribution* of the responses of the GAM to the CS is distinguishable from the *distribution* of responses to the non-learned stimuli. This notion becomes exact in the next section.

### The capacity of the GAM

How many CS can be stored in a GAM? Addressing this question using the full dynamical equations (Figure 4) requires extensive simulations that are beyond the scope of this paper. Therefore, we use a binary approximation (see Materials and Methods). The quality of the binary approximation is demonstrated in Figure S1 in the Supporting Information.

**Figure 5:**
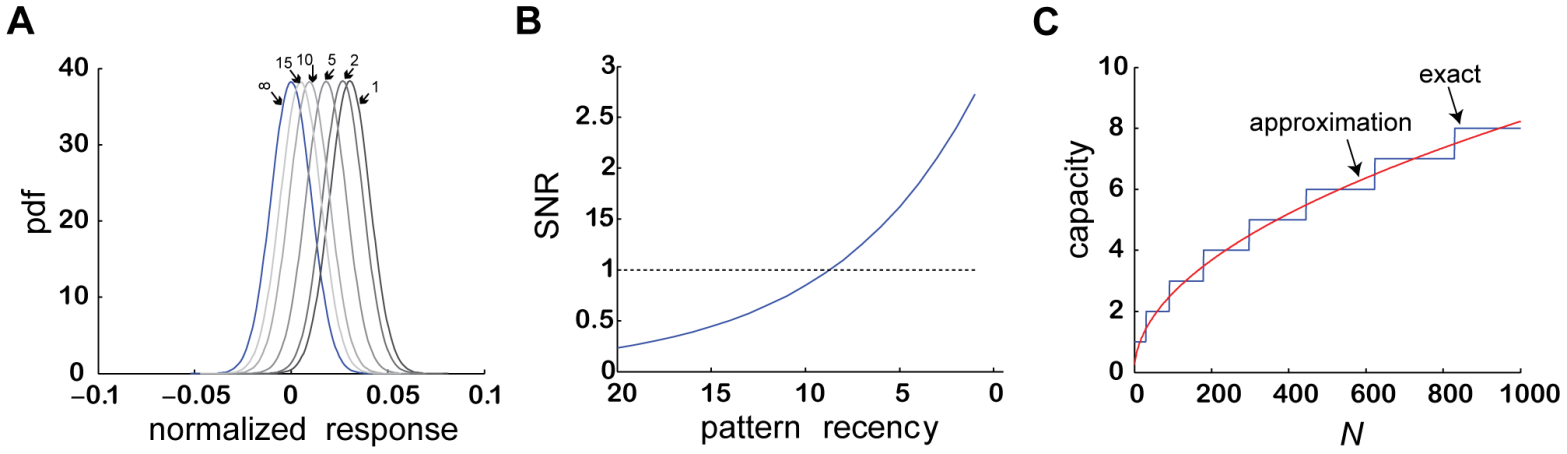
The capacity of a single GAM to maintain associations. (A) a distribution plot of the normalized response, 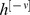, as a function of the age of the CS. (B) the SNR as a function of the age of the CS. 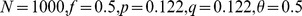 and 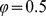. (C) The capacity of the GAM to store memories as a function of *N*. Blue, exact Markov model; Red, approximated model (Eq. 20).

As described in the previous section, responses to non-learned stimuli depend on the overlap of the pattern of activation of the stimuli with the pattern of activation of the pseudo-synapses. Because both the stimuli and the dynamics of the pseudo-synapses are stochastic, this response is a stochastic variable. The distribution of the responses to non-learned stimuli (see Eq. (14) in the Materials and Methods) is depicted in Figure 5A (blue). The response of the GAM to learned CS is also a stochastic variable. The distribution of responses to the most recently learned CS is depicted in Figure 5A (black; Eq. (13) and in the Materials and Methods). This distribution is well-separated from the distribution of responses to the non-learned stimuli. Therefore, recently-learned stimuli are distinguished from non-learned stimuli using a simple threshold mechanism (e.g., the dashed line in Figure 4B). The probability of an error depends on the overlap between the two distributions. If the overlap is small, the GAM almost always responds to the most recently learned CS and almost never responds to non-learned stimuli. On the other hand, a large overlap would result in a large number of errors, false positives or misses, depending on the choice of threshold. The difference between the means of the two distributions (black and blue) depends on the transition probabilities. The higher the probabilities, the larger the difference is. Therefore, the higher the transition rates are, the easier it is to distinguish between the most recently learned CS and the non-learned stimuli.

The distribution of responses to the presentation of the second-most recently learned CS (darkest gray) is also to the right of the distribution of responses to non-learned stimuli (blue). Nevertheless, it is shifted to the left relative to the distribution of responses to the most recently learned CS (black). As a result, the overlap of this distribution with the distribution of responses to the non-learned stimuli is larger. The reason for this shift is that as noted in Figure 4B, the newer CS ‘overwrites’ the memory of the older CS, resulting in a decreased overlap between the CS and the pseudo-synapses. The degree of overwriting, manifested as a shift to the left of the distribution of responses to the second-most recently learned CS relative to the most recently learned CS, depends on the US-induced transition rates. The smaller the transition rates, the smaller the overwriting is and therefore the smaller the shift to the left of the distribution.

More generally, the distributions of responses to a CS shift to the left with the ‘age’ of the CS. This is depicted in Figure 5A using grayscale. While the distribution of the several most-recently learned CS is well-separated from the distribution of responses to non-learned stimuli (blue in Figure 5A), the distributions of responses to ‘older’ CS and non-learned stimuli largely overlap, indicating that ‘older’ CS are ‘forgotten’.

More formally, the ability of the GAM to distinguish between a learned CS and a non-learned stimulus depends on the signal to noise ratio (SNR), which is defined as the ratio of the difference in mean responses to the two classes of stimuli, divided by the square root of the sum of variances of the two distributions (Eq. (14) in the Materials and Methods). In general, the larger the SNR, the fewer errors when distinguishing between learned and non-learned stimuli. The SNR, as a function of the ‘age’ of the CS is depicted in Figure 5B: the newer the CS, the larger the SNR. The SNR of the 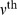 CS (where the numbering of patterns is reversed such that 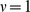 corresponds to the most recent stimulus) is given by Eq. (14) in the Materials and Methods section. The capacity of the GAM can thus be defined as the ‘oldest’ CS such that the corresponding SNR is larger than 1. In other words, the capacity of the GAM 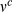 is defined as the largest value of 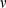 such that 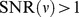.

The capacity of the GAM depends on the US-induced transition rates, which determine the transition probabilities. As discussed above, if these rates are high, forgetting is fast. On the other hand, if these rates are too low the GAM cannot reliably retrieve even the most recent CS. The capacity of the GAM is maximal when the US-induced transition rates are intermediate, balancing between these two requirements. The capacity of the GAM as a function of the number of pseudo-synapses (*N*) is depicted in Figure 5C (blue). The larger *N*, the larger is the capacity of the GAM. In the Materials and Methods section we show that in the limit of 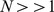, if the US-induced transition probabilities are optimal, the capacity of the GAM is proportional to the square root of the number of pseudo-synapses, 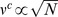 (Eq. (20); red line in Figure 5C). This result is similar to the memory capacity of models of neural networks with binary synapses [47], [48]. However, the learning rule proposed here, even in the binary approximation, differs from the Hebbian synaptic plasticity rule used in neural network models [47], [48].

### The wisdom of crowds

In the previous sections we studied the ability of a single GRN to learn associations. However in nature, GRNs often do not reside in isolation but in populations comprising of a large number of individual cells of the same type, e.g., as in a colony of bacteria or in a tissue, all exposed to the same external conditions. This raises an interesting question: is the capacity of a population of GAMs to store associations larger than that of a single GAM? The answer is trivially positive if we allow the different GAMs to communicate and form a recurrent network with specialized connections between individual GAMs, similar to neurons in neuronal networks. However, here we ask a different question: is the capacity of a population of non-interacting GAMs to store and retrieve memories different from that of the single GAM?

We consider a population of generalized GAMs as in Figure 4A. All GAMs are identical, exposed to the same sequence of stimuli but differ in their internal stochasticity. In other words, the noise 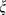 associated with the dynamics of the pseudo-synapses (Eq. (2)) in the different GAMs is assumed to be independent. The population response in our model is assumed to be simply the accumulated response of all individual GAMs.

**Figure 6:**
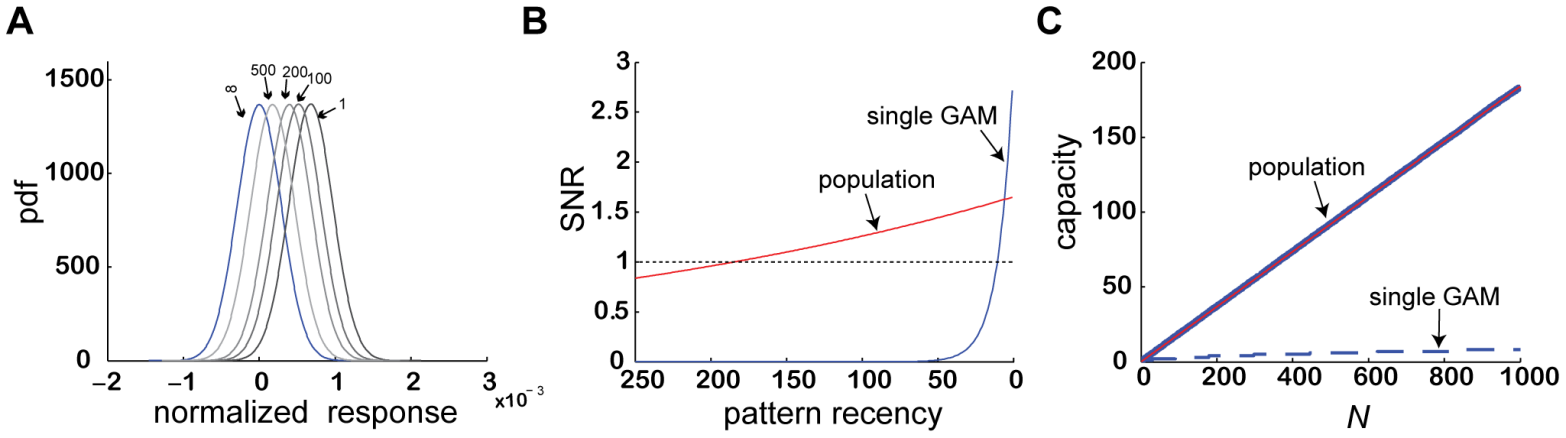
The capacity of a large population of GAMs to maintain associations. (A) a distribution plot of the normalized response, 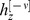 , as a function of the age of the CS. (B) Solid blue line, the SNR of a population of GAMs as a function of the age of the CS. 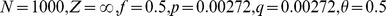, and 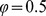. Dashed blue line, the single GAM, same as in Figure 5B. (C) The capacity of a population of GAMs as a function of *N*. Blue line, exact Markov model; Red line, approximated model (Eq. 20). Note that the blue and red lines almost overlap. Dashed blue line, the single GAM, same as in Figure 5C.

In order to understand why the capacity of a population of identical GAMs to store memories may be larger than the capacity of a single GAM, we note that a CS of a particular ‘age’ can be retrieved if the overlap between the distributions of responses to the learned and non-learned stimuli is sufficiently small. This overlap is sensitive to the variances of the two distributions (width of the curves in Figure 5A). The larger the variance, the larger is the overlap. Two sources contribute to this variance in the responses. First, there is stochasticity in the realization of CS and non-learned stimuli. Second, there is stochasticity in the encoding process. While the first type of stochasticity is external and thus shared by all GAMs in the population, the second type of stochasticity is independent for each GAM. As a result, when considering the cumulative response of a large population of GAMs, all other parameters being equal, the variance in the distribution of responses is considerably smaller (Eq. (23) in the Materials and Methods). In Figure 6A we plot the distributions of responses to CS of different ‘ages’ (gray, color-coded) and non-learned stimuli (blue).

Similar to the case of a single GAM, the capacity of a population of GAMs depends on the US-induced transition rates. However, because the variance in the responses in the case of the population is considerably smaller than the variance in the case of a single GAM, the US-induced transition probabilities that maximize the capacity of the population are considerably smaller than those that maximize the capacity of a single GAM. In Figure 6B we plot the SNR as a function of the ‘age’ of the CS (solid blue line). Compared to the SNR of a single GAM (dashed blue line, identical to Figure 5B), the SNR of the response of the population of GAMs is larger than 1 for much ‘older’ CS.

The capacity of the population of GAMs as a function of the number of pseudo-synapses (*N*) is depicted in Figure 6C (solid blue line). The larger *N* is, the larger is the capacity of the GAMs. More quantitative analysis reveals that for an appropriate choice of parameters, the number of different CS that a large population of GAMs can store is proportional to the number of pseudo-synapses (Eq. (31); solid red line), compared to a capacity that is only proportional to the square root of the number of pseudo-synapses in the case of a single GAM (dashed blue line, identical to Figure 5C).

## Discussion

In this paper, we explored the ability of a general GRN to encode associations. We showed that a GRN that is endowed with bistable elements and stochastic dynamics is capable of storing and retrieving multiple arbitrary and overlapping associations. The capacity of a single GRN in our model, defined as the number of stored associations, is proportional to the square root of the number of bistable elements 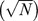. This result is reminiscent of Hopfield-like models with bounded synapses, in which the capacity is proportional to the square root of the number of synapses [47], [48], [49]. Remarkably, in a large population of GRNs, as is in a colony of bacteria or in a tissue, this capacity is substantially higher and is proportional to the number of bistable elements.

Despite the similarities between the GAM and the Hopfield model, there are two important differences that are noteworthy. First, the capacity of a single GAM may be limited by the presence of readout noise (e.g., in the dynamics of *R*). However, this readout noise is not expected to substantially affect the capacity of a population of GAMs because of averaging. Second, the number of neurons available in neuronal networks is much larger than the number of bistable elements in GRNs. Altogether, our model predicts that if the number of bistable elements in the GRN does not exceed several tens, it will be difficult to store more than one or two memories in a single GAM. Therefore, the storage of multiple memories is likely to require a population of GAMs.

The key elements in our model are the bistability and the stochasticity of the dynamics of the GRN. Importantly, bistability and stochasticity are not restricted to the transcriptional machinery. Rather, they are found in various cellular processes, including post-transcriptional regulation (e.g., by non-coding RNA [50], [51]) or post-translational regulation (e.g. phosphorylation and degradation regulation [36], [52], [53], [54]). We modeled associative memory that is based on the interaction of proteins through the transcriptional machinery because these dynamics are better characterized and are more accessible experimentally than other cellular alternatives.

Moreover, the GAM is not restricted to a particular organism. The parameters used in the simulations presented in this paper are biologically plausible for bacteria. However, because the basic elements of the GAM, namely, bistability and stochasticity, are widespread in GRNs of all cells, the potential for associative learning without a nervous system exists for virtually all cell types, including single-celled eukaryotes and plants. Furthermore, this work suggests that even in animals that possess a nervous system, learning that is independent of this nervous system is also possible. In particular, it could be interesting to consider GAM in the immune system, which has evolved to learn to respond to novel pathogens.

Bearing this in mind, we believe that in view of the recent developments of experimental methods that quantitatively measure the expression level of proteins, bacteria, in particular the well characterized *E. coli*, are the ideal substrate to study the associative learning in GRNs. Each of the components of the GAM module (Figure 3A), namely inducible elements, bistable switches and AND gates, have been established in the *E. coli* transcription network and therefore a synthetic implementation is achievable [55], [56].

Beyond synthetic implementation, the complexity of the genetic networks suggests that GAM-like modules may exist. A first step in searching for GAMs in known networks should be the identification of plausible candidates for the US, UR and CS. In animals, the US is a stimulus that causes an overt response prior to learning, the UR. Typically the US is a stimulus of biological significance, such as food or a noxious stimulus and the UR is an ecologically-relevant overt response, often in the form of muscle activation. For example, in the eye-blink conditioning experiment (Figure 1) the US is an air puff and the UR is an eye blink that protects the eye from the puff. An important point to consider when searching for associative learning in bacteria is ecological significance. Our model for associative learning, similar to models of associative learning in neuronal networks, does not incorporate any ecological information about the stimuli. However in animals, it is known that the ability to form an association depends on the ecological relevance of the CS to the US. For example, the association of the taste of a certain food (CS) with the symptoms caused by a toxic or spoiled food (US), known as taste aversion, is easily-formed after a small number of repetitions. By contrast, it is substantially more difficult to form an association of a tone with the same US [57]. It is generally believed that this difference results from the fact that typically, taste is more informative about the chemical composition of substances than auditory signals. Therefore, taste-aversion but not tone-aversion has evolved as a specific learning mechanism aimed at preventing the consumption of poisonous substances. Drawing an analogy to associative learning in bacteria, we propose to utilize ecologically-relevant CS rather than arbitrary CS when searching for associative learning in bacteria. In our model, the strength of association increases with the number of repetitions due to the stochasticity in the encoding process. Such dependence of the strength of association on the number of repetitions is also observed in classical conditioning experiments in animals [58]. Therefore, experiments involving a large number of co-occurrences of the CS and US are more likely to reveal associative learning in GRNs or populations of GRNs. Note that standard experiments studying responses of bacteria are typically short and do not involve repetitions in the presentation of stimuli to the same population of bacteria. Therefore, associative learning in such experiments may have been overlooked. Moreover, we have shown that the learning capacity of the population of bacteria is higher than that of a single GRN. Therefore, the experimental search for associative learning in bacteria should be done at the population level.

More specifically in bacteria, the presence of foreign bacteria is a signal of potential stress. For example, many bacteria produce antibiotics that are harmful to other strains [59]. Other bacteria are sensitive to these damaging antibiotics and respond to their presence by activating a pre-wired stress response, such as the multiple antibiotics response (MAR) [60]. We thus suggest that the *R* gene in our scheme corresponds to one of the outputs of MAR response, e.g. the micF gene [61]. Note that similar to the blink in the classic eye-blink conditioning that protects the eye from the air puff (Figure 1), the activation of micF prevents the entry of the antibiotics into the cell. Thus, the antibiotics can be considered as a US whereas the stress response can be considered as a UR. However, the production of harmful antibiotics is not present in all bacteria species. Therefore, learning to distinguish between harmful and benign strains of bacteria is of potential great ecological significance because it may allow the bacteria to respond faster. Thus, the presence of foreign bacteria could correspond to the CS in our framework. Indeed, bacteria are able to detect secondary metabolites that are produced by other strains [62]. In that line, we suggest as a candidate for the *M* protein in the model the MarA gene. MarA is known to positively autoregulate itself, and thus has the potential to be bistable. In addition, the promoter of that gene contains multiple binding sites for transcription factors, allowing for complex regulation of the gene expression including the realization of AND gates.

Experimentally, the UR can be measured using a fluorescent-based reporter that is regulated by a promoter of a stress response gene. The CS in this framework should be stimuli that can be sensed by the bacteria but do not elicit the stress response. These include a change in the concentration of different molecules that does not activate the stress response. Repeated exposure to such conditions can be controlled using a chemostat [63], which can maintain selected growth conditions at a constant level while changing others.

Finally, the benefit of the stress response at the population level can also be found in the induction of the MAR response, as it triggers the activation of genes that inactivate toxic compounds. The benefit of this “pooled response” for the population comes from the decrease in the concentration of the toxic compound [64].

Whether or not associative learning exists in GRNs on a time-scale much shorter than required for evolution is an open question. However, whether considering bacteria that can predict a stress condition or human digestive cells that can predict food intake, associative learning in single and populations of cells seems to have an evolutionary advantage. In view of the computational capabilities of GRNs demonstrated in this paper, we believe that future careful investigations will reveal the existence of associative learning in single and populations of cells.

## Materials and Methods

### The dynamical equations of the generalized model

In this section we provide the rate equations that describe the dynamics of the generalized model (Figure 4A). The kinetic reactions that underlie these dynamics and the derivation of the rate equations from the kinetic reactions are described in Text S1 in the Supporting Information. The single pathway equations correspond to *the* generalized model with 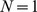.

The equations that describe the dynamics of the pseudo-synapses are given by:
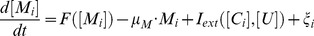
(4)
*where* 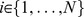. The nonlinear positive feedback term, 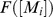, is described by 
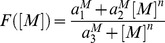
 where the parameters 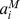 are kinetic parameters, and *n* is the Hill coefficient, corresponding to the cooperativity of binding. The second term in Eq. (4) denotes the protein degradation, where 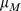 is a parameter. The third term in Eq. (4) describe the effect of 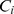 and *U*, 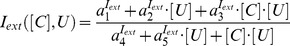 where 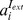 are kinetic parameters. The last term in Eq. (4) models the stochasticity of the dynamics, and we assume that 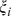 are independent white noise such that 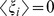, 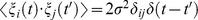 where 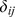 is Kronecker's delta function such that 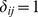 if 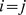 and 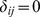 otherwise and 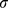 is a parameter.

The reactive equation that describes the dynamics of *R* is given by:
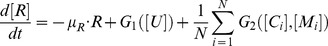
(5)where 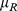 is the degradation rate of *R*, 
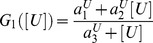
 and 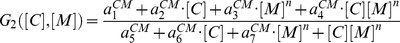 where 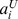 and 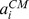 are kinetic parameters.

### The capacity of the GAM

In this section we compute the capacity of the GAM to learn associations. To that goal, we consider a binary approximation of the dynamics of the pseudo-synapses. Because the dynamics of *M* spend most of the time near the attractors of the deterministic dynamics, Eq. (1), it can be approximated using a two-state Markov chain, where each state corresponds to one attractor of the deterministic dynamics. We further assume that the US and CS are presented in discrete “trials” composed of a fixed period of time. Therefore, the response of *M* to the presentation of the CS and US can be approximated by:
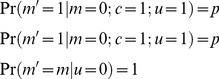
(6)where 
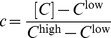
 and 
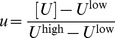
 such that 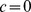 and 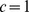 denote epochs in which 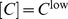 and 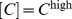, respectively and 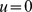 and 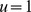 denote epochs in which 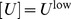 and 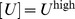, respectively. The variables *m* and *m′* denote the states of the pseudo-synapse before and after the presentation of the external cues and their values; 0 or 1 denote epochs in which 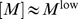 and 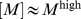, respectively. The steady state response to the presentation of a pattern is:
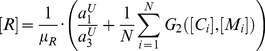
(7)The selectivity of the response in Eq. (7) depends on the value of the sum of 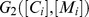. In response to the presentation of a CS that was learned 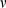 CSs ago,
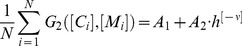
(8)where
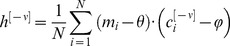
(9)

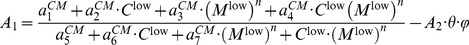


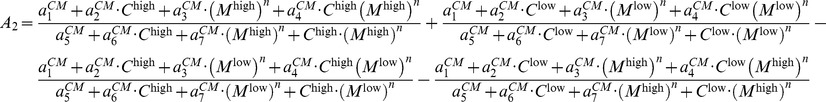
and
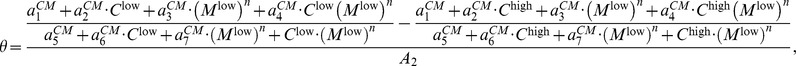


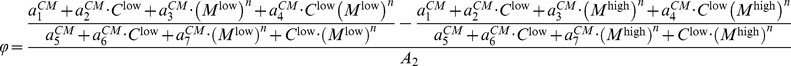
(see Text S1 in Supplementary Information for a more detailed derivation).

Dissociating a learned pattern 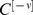 from non-learned patterns (which we denote as 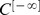) is possible only if 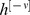 is significantly different from 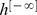. The difficulty in dissociating learned and non-learned patterns lies in the fact that the responses to the two types of patterns are stochastic variables that depend on the stochasticity in the realization of the learned and non-learned stimuli as well as the stochasticity in the learning. Therefore, we consider the distribution of responses to the learned and non-learned stimuli.

To compute the distribution of 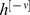, note that in response to the presentation of a sequence of CS, changes in the state of the pseudo-synapses follow a Markov chain such that
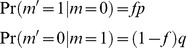
(10)From Eq. (10) it follows that at the stationary distribution,
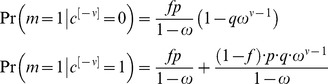
(11)where 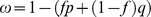.

Using Eq. (11), and the fact that 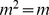 and 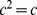, a straightforward calculation yields that the mean and variance of 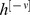 are given by:
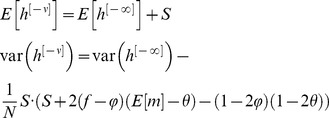
(12)where
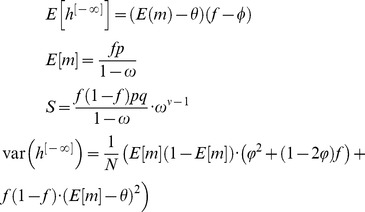
(13)Note that for large *N* 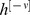 is the sum of a large number of independent and identically distributed random variables and therefore according to the central limit theorem 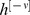 is normally distributed.

In order to compute the capacity of the GAM, we define the difference between the mean responses to learned and non-learned stimuli as the signal and the square root of the sum of the variances of the responses to the learned and non-learned stimuli as the noise. In the limit of large *N*, the ability of a binary classifier to discriminate between the learned and non-learned stimuli depends on the SNR. If the SNR is large, it is possible to achieve a high detection rate while maintaining a low level of false positives. A low SNR implies that the two stimuli are indistinguishable. Therefore, we define the capacity of the GAM to be the oldest memory such that the SNR is larger than 1 (see also [47], [48] for a similar approach in models of neural networks). Formally, the signal-to-noise-ratio for a pattern presented 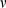 patterns ago is given by:
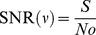
(14)where
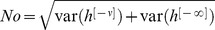
(15)We compute the capacity in the limit of large *N* and consider the effect of the scaling of *p* and *q* with *N* on the capacity of the GAM. If the values of *p* and *q* are very different then the pseudo-synapses will saturate. Therefore, we consider the same scaling of *p* and *q*, 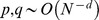. The signal in Eq. (13) depends on the product of two terms, 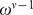 that depends on 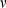 and a prefactor, 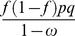 that is independent of 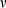. It is easy to see that the prefactor, 
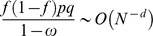
. Similarly, it is easy to see that 
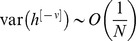
. Therefore, 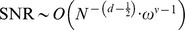. Because 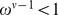, a necessary condition for the SNR to be larger than 1 is 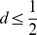. The term 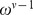 decays exponentially fast with 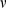. However, because 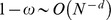, as long as 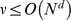, 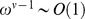. Therefore, for 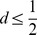, as long as 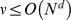, 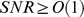. Thus, for 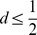, the capacity of the GAM is 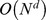, which is maximal for 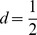. In other words, assuming that 
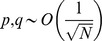
, the capacity of the GAM is 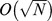.

To gain insights into this result, we consider the optimal choice of 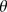 and 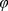 (which minimizes the variance), in which 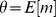 and 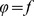. In this case, in the limit of large *N*,
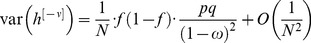
(16)and Eq. (15) becomes
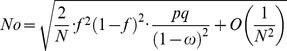
(17)Thus, Eq. (14) becomes:
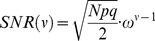
(18)The requirement that 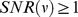 yields:
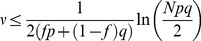
(19)where we used the fact that for 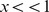, 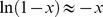 and therefore 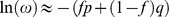.

In order to find the values of 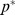 and 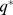 that maximize the capacity of the GAM, we compute the zeros of the partial derivatives of Eq. (19) with respect to 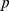 and 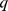, resulting in 
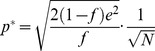
 and 
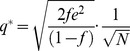
. Thus, the capacity of the GAM is
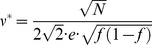
(20)For 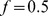, the capacity is 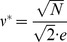. Note that the capacity increases as the value of *f* deviates from 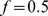.

### The capacity of a population of GAMs

In this section we compute the capacity of a large population composed of *Z* identical GAMs. The population response to the presentation of 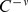 is given by (up to constant shift and scaling):
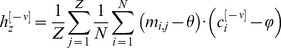
(21)where 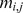 is the 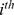 pseudo synapse (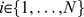) in the 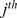 GAM (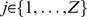) (compare to Eq. (9)).

Similar to the analysis of the capacity of a single GAM, we compute the mean and variance of 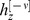. The computation of mean of 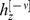 in the case of the population is similar to that computation for the case of a single GAM, yielding
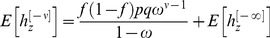
(22)Note that 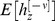 is independent of the size of the population: since all GAMs are identical, their contribution, on average, is equal. Computing the variance of Eq. (21) results yields:
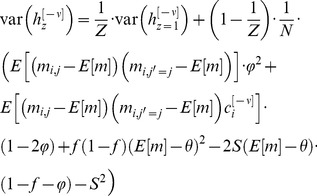
(23)In order to evaluate Eq. (23), we consider the dynamics of a single pseudo-synapse 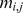. Similar to Eq. (3),
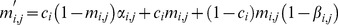
(24)where 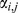 and 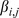 are Bernoulli variables with parameters *p* and *q*, respectively.

Using induction, it is easy to prove that the value of 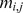 in response to the learning of an infinite sequence of CS is given by:
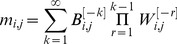
(25)where 
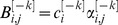
 and 
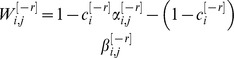
 and 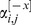 and 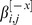 denote the values of the Bernoulli variables 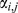 and 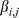, respectively, during the encoding of the CS *x* patterns ago.

Using Eq. (24), it can be shown that:
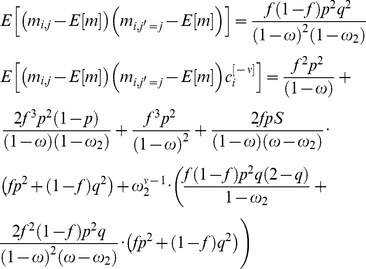
(26)where 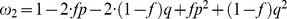.

Substituting Eq. (26) in Eq. (23) and assuming that 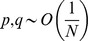 yields:
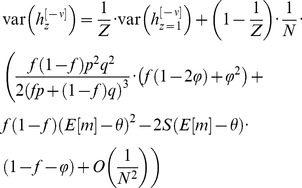
(27)Note that in the case of a single network, 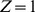, only the first term contributes, yielding Eq. (16).

The capacity of the population of GAMs is defined as the oldest memory such that the SNR is larger than 1, where the signal and noise terms in Eq. (14) are given by
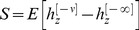
(28)And
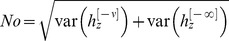
(29)In the limit of 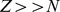 (large number of GAMs) the contribution of the first term in Eq. (26) to the variance vanishes and the capacity depends on the second term. For a general value of 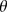, 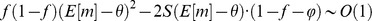 and this term dominates 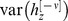, resulting in 
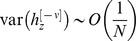
. Therefore, the population capacity in this case is 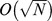, similar to that of a single GAM. However, if 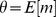 this 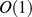 term vanishes and 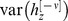 is dominated by 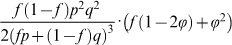, resulting in 
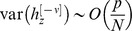
. Similar to the case of a single GAM, we compute the capacity in the limit of large *N* and consider the effect of the scaling of *p* and *q* with *N* on the capacity of the population of GAMs. If the values of p and q are very different then the pseudo-synapses will saturate. Therefore, we consider the same scaling of *p* and *q*, 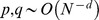. The signal in Eq. (27) is the same as that of a single GAM (Eq. (13)), therefore the prefactor in Eq. (13) is 
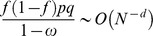
. Similarly, it is easy to see that 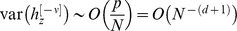. Therefore, 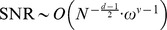. Because 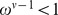, a necessary condition for the SNR to be larger than 1 is 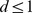. The term 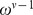 decays exponentially fast with 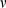. However, because 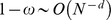, as long as 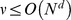, 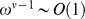. Therefore, for 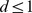, as long as 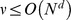,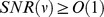. Thus, for 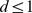, the capacity of the GAM is 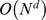, which is maximal for 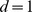. In other words, assuming that 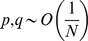, the capacity of the GAM is 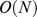. In particular, assuming that 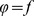, substituting Eqs. (28) and Eq. (29) in Eq. (14) yields
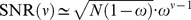
(30)A straightforward calculation reveals that the capacity is maximal when 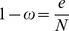, resulting in capacity:
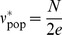
(31)


### Numerical procedures

In our simulations, we used the following parameters: 
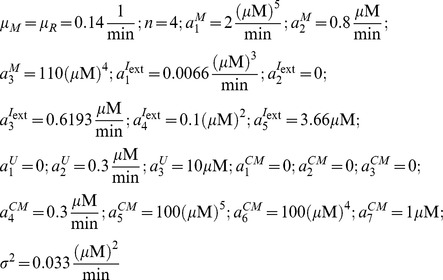
For the generalized model (Figure 3) we used: 
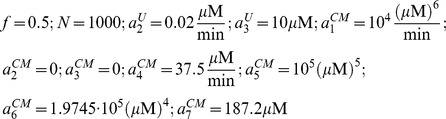
All other parameters were the same as the single pathway model (Figure 3). The derivation of the parameters from the reaction kinetic constants is provided in the Text S1 in the Supporting Information. The reaction kinetic constants that were used are provided in Table S1 in the Supporting Information. Simulations in Figures 3 and 4 were carried out using Euler method for numerical integration with step sizes 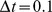
and 0.5 min, respectively.

## Supporting Information

Figure S1 **Comparing the dynamics equations and the Markov approximation.** (A) Green line, the response *R* in a simulation of the model (Eqs. (4) and (5)) in the same paradigm as in Figure 4B. Orange rectangles, the timing of a US; Letters A–E denote the timing as identities of CS. (B) The responses *R* to patterns A–E prior to learning (blue, green, black, magenta and yellow lines, respectively) and to pattern A after learning (red line), at the times corresponding to the corresponding colored horizontal lines in A, aligned to the time of presentation of the stimuli. Circles, mean response in the second half of stimulus presentation (last 15min) for each pattern. . (C) Histograms of mean responses (circles in B) to the most recently learned patterns (black) and random patterns (blue). (D) The SNR as a function of the age of the pattern. Red circles, the dynamics equations, blue line, the predicted SNR from the Markov model. Green line, the predicted SNR assuming optimal parameters. Note that the green and blue lines almost overlap, and that they both agree well with the simulation red circles. The parameters that were used in the simulation are the same as those used in Figure 4B and the SNR and histograms are based on 1,000 repetitions.
(TIF)

Table S1 **Kinetic parameters used in simulations.** A table of all the kinetic parameters used to derive the parameters of the numerical simulations of the approximate dynamics.
(PDF)

Text S1 **Derivations of the approximate dynamic equations** **(Eqs. 1** **–** **9)** **from the kinetic reaction equations**
(PDF)
