# Supplementary figures and images for: Stochasticity, Bistability and the Wisdom of Crowds: A Model for Associative Learning in Genetic Regulatory Networks
Source: PLoS Comput Biol. 2013 Aug 22;9(8):e1003179. doi: 10.1371/journal.pcbi.1003179 (PMC3749950; doi:10.1371/journal.pcbi.1003179)

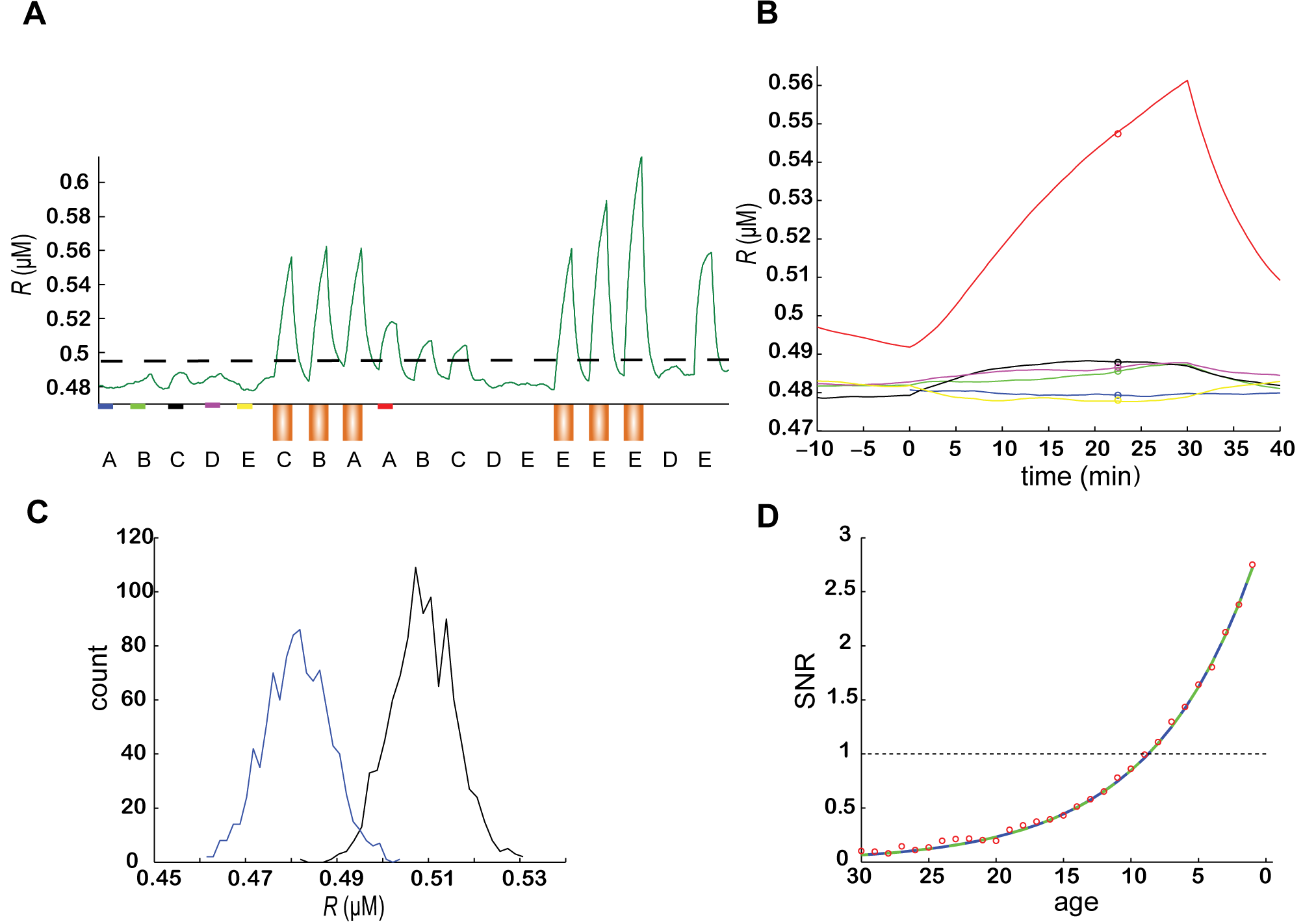

Supplement: Figure S1 — Comparing the dynamics equations and the Markov approximation. (A) Green line, the response R in a simulation of the model (Eqs. (4) and (5)) in the same paradigm as in Figure 4B. Orange rectangles, the timing of a US; Letters A–E denote the timing as identities of CS. (B) The responses R to patterns A–E prior to learning (blue, green, black, magenta and yellow lines, respectively) and to pattern A after learning (red line), at the times corresponding to the corresponding colored horizontal lines in A, aligned to the time of presentation of the stimuli. Circles, mean response in the second half of stimulus presentation (last 15min) for each pattern. . (C) Histograms of mean responses (circles in B) to the most recently learned patterns (black) and random patterns (blue). (D) The SNR as a function of the age of the pattern. Red circles, the dynamics equations, blue line, the predicted SNR from the Markov model. Green line, the predicted SNR assuming optimal parameters. Note that the green and blue lines almost overlap, and that they both agree well with the simulation red circles. The parameters that were used in the simulation are the same as those used in Figure 4B and the SNR and histograms are based on 1,000 repetitions. (TIF) [file pcbi.1003179.s001.tif]
