# Supplementary material for: Stochasticity, Bistability and the Wisdom of Crowds: A Model for Associative Learning in Genetic Regulatory Networks
Source: PLoS Comput Biol. 2013 Aug 22;9(8):e1003179. doi: 10.1371/journal.pcbi.1003179 (PMC3749950; doi:10.1371/journal.pcbi.1003179)
Supplement: Table S1 — Kinetic parameters used in simulations. A table of all the kinetic parameters used to derive the parameters of the numerical simulations of the approximate dynamics. (PDF) [file pcbi.1003179.s002.pdf]

SUPPORTING INFORMATION

Stochasticity, Bistability and the Wisdom of Crowds: a Model for Associative Learning in Genetic Regulatory Networks

Matan Sorek, Nathalie Q. Balaban and Yonatan Loewenstein

Table S1 - Kinetic Parameters used in simulations

A table of all the kinetic parameters used to derive the parameters of the numerical simulations of the approximate dynamics.

The single pathway model:

**Parameter**	**Value**
$[P_T^1]$	$1\ nM$
$[P_T^2]$	$1\ nM$
$[P_T^{R,1}]$	$1\ nM$
$[P_T^{R,2}]$	$1\ nM$
n	4
μ_R	$0.1388\ \frac{1}{min}$
μ_M	$0.1388\ \frac{1}{min}$
$\frac{\beta}{\delta}$	100
$\frac{\beta_R}{\delta_R}$	100
α_1	$0.1818\ \frac{1}{min}$
α_2	$8\ \frac{1}{min}$
α_3	$0.658\ \frac{1}{min}$
α_4	$6.1926\ \frac{1}{min}$
K_1^{-1}	$110\ (\mu M)^4$
K_2^{-1}	$0.0273\ \mu M$
K_3^{-1}	$3.66\ \mu M$
α_1^R	$0\ \frac{1}{min}$
α_2^R	$3\ \frac{1}{min}$
α_3^R	$0\ \frac{1}{min}$
α_4^R	$0\ \frac{1}{min}$
α_5^R	$0\ \frac{1}{min}$
α_6^R	$3\ \frac{1}{min}$

$K_{1,R}^{-1}$	$1\ \mu M$
$K_{2,R}^{-1}$	$1\ \mu M$
$K_{3,R}^{-1}$	$100\ (\mu M)^4$
$K_{4,R}^{-1}$	$100\ (\mu M)^4$

The generalized model:

**Parameter**	**Value**
α_1^R	$0\ \frac{1}{min}$
α_2^R	$0.2\ \frac{1}{min}$
α_3^R	$0.001\ \frac{1}{min}$
α_4^R	$0\ \frac{1}{min}$
α_5^R	$0\ \frac{1}{min}$
α_6^R	$0.375\ \frac{1}{min}$
$K_{1,R}^{-1}$	$10\ \mu M$
$K_{2,R}^{-1}$	$0.5064\ \mu M$
$K_{3,R}^{-1}$	$534.2\ (\mu M)^4$
$K_{4,R}^{-1}$	$19745\ (\mu M)^4$
f	0.5

All other parameters of the generalized model are the same as for the single pathway model.
