# Supplementary material for: Stochasticity, Bistability and the Wisdom of Crowds: A Model for Associative Learning in Genetic Regulatory Networks
Source: PLoS Comput Biol. 2013 Aug 22;9(8):e1003179. doi: 10.1371/journal.pcbi.1003179 (PMC3749950; doi:10.1371/journal.pcbi.1003179)
Supplement: Text S1 — Derivations of the approximate dynamic equations (Eqs. 1 – 9) from the kinetic reaction equations (PDF) [file pcbi.1003179.s003.pdf]

SUPPORTING INFORMATION

Stochasticity, Bistability and the Wisdom of Crowds: a Model for Associative Learning in Genetic Regulatory Networks

Matan Sorek, Nathalie Q. Balaban and Yonatan Loewenstein

Text S1 - Derivations of the approximate dynamic equations (Eqs. 1-9) from the kinetic reaction equations

In this section we derive the approximate dynamics of the pseudo-synapses *M* and the response protein *R* from the kinetic reaction equations. The section is divided into four parts: (1) the derivation of Eq. (1); (2) the derivation of Eq. (3); (3) the derivation of Eq. (5), and (4) the derivation of Eqs. (8-9).

1. Derivation of Eq. (1) with I_{ext} that depends on C and U

We assume that the expression of *M* is regulated by two promoters, denoted by P_M^1 and P_M^2. The promoter P_M^1 mediates the self-regulation of *M* such that its dynamics follow the transcription factor (TF)-DNA binding kinetics:

(S1) $$P_M^1 + nM \underset{k_{-1}}{\overset{k_1}{\rightleftharpoons}} P_M^1 M_n$$

where k_1 and k_{-1} are the on and off rate parameters, respectively. The corresponding kinetics are:

(S2) $$\frac{d[P_M^1 M_n]}{dt} = k_1[P_M^1][M]^n - k_{-1}[P_M^1 M_n]$$

where $[x]$ denotes the concentration of x, with the conservation equation

(S3) $$[P_T^1] = [P_M^1] + [P_M^1 M_n]$$

The promoter P_M^2 is regulated by protein *U*, which signals the US:

(S4) $$P_M^2 + U \underset{k_{-2}}{\overset{k_2}{\rightleftharpoons}} P_M^2 U$$

In addition, the complex $P_M^2 U$ is regulated by *C*, which signals the CS:

(S5) $$P_M^2 U + C \underset{k_{-3}}{\overset{k_3}{\rightleftharpoons}} P_M^2 UC$$

The kinetics corresponding to Eqs. (S3) and (S4) are

(S6) $$\frac{d[P_M^2 U]}{dt} = k_2[P_M^2][U] + k_{-3}[P_M^2 UC] - k_{-2}[P_M^2 U] - k_3[P_M^2 U][C]$$

(S7) $\frac{d[P_M^2UC]}{dt} = k_3[P_M^2U][C] - k_{-3}[P_M^2UC]$

with the conservation equation

(S8) $[P_T^2] = [P_M^2] + [P_M^2U] + [P_M^2UC]$

The two promoters P_M^1 and P_M^2 regulate the synthesis of *M*'s mRNA, m_M, which is translated into protein *M*. The synthesis and degradation equations of m_M are given by:

(S9) $P_M^1 \overset{\alpha_1}{\rightarrow} m_M$

(S10) $P_M^1 M_n \overset{\alpha_2}{\rightarrow} m_M$

(S11) $P_M^2 \overset{\alpha_3}{\rightarrow} m_M$

(S12) $P_M^2 U \overset{\alpha_4}{\rightarrow} m_M$

(S13) $P_M^2 UC \overset{\alpha_5}{\rightarrow} m_M$

(S14) $m_M \overset{\delta}{\rightarrow} \phi$

The kinetics corresponding to Eqs. (S9)-S(14) are

(S15) $\frac{d[m_M]}{dt} = \alpha_1[P_M^1] + \alpha_2[P_M^1 M_n] + \alpha_3[P_M^2] + \alpha_4[P_M^2U] + \alpha_5[P_M^2UC] - \delta \cdot m_M$

The production and degradation of the protein *M* are given by:

(S16) $m_M \overset{\beta}{\rightarrow} M$

(S17) $M \overset{\mu_M}{\rightarrow} \phi$

with the resulting kinetics

(S18) $\frac{d[M]}{dt} = \beta \cdot [m_M] - \mu_M \cdot [M]$

In order to derive Eq. (1), we assume that $[M] \gg [P_M^1]$ and $[C],[U] \gg [P_M^2]$ and therefore, the total concentration of the bound and unbound proteins *M*, *C* and *U* ($[M_T]$, $[C_T]$ and $[U_T]$, respectively) are approximately equal to the concentration of the unbound proteins ($[M]$, $[C]$ and $[U]$, respectively). Formally,

(S19) $[M_T] = [M] + n \cdot [P_M^1 M_n] \approx [M]$

(S20) $[C_T] = [C] + [P_M^2UC] \approx [C]$

(S21) $[U_T] = [U] + [P_M^2U] + [P_M^2UC] \approx [U]$

With these assumptions, the steady state solutions of Eqs. (S2), (S6) and (S7) are given by:

(S22) $[P_M^1] = \frac{[P_T^1]K_1^{-1}}{K_1^{-1}+[M]^n}$

(S23) $[P_M^1 M_n] = \frac{P_T^1[M]^n}{K_1^{-1}+[M]^n}$

(S24) $[P_M^2] = \frac{[P_T^2]K_2^{-1}K_3^{-1}}{K_2^{-1}K_3^{-1}+K_3^{-1}[U]+[C][U]}$

(S25) $[P_M^2 U] = \frac{[P_T^2]K_3^{-1}[U]}{K_2^{-1}K_3^{-1}+K_3^{-1}[U]+[C][U]}$

(S26) $[P_M^2 UC] = \frac{P_T^2[C][U]}{K_2^{-1}K_3^{-1}+K_3^{-1}[U]+[C][U]}$

Assuming that the time-scales of the TF-DNA bindings (Eqs. (S2), (S6) and (S7)) are much shorter than the time scale of mRNA synthesis and degradation (Eq. (S15)), we substitute Eqs. (S22)-(S26) in Eq. (S15). The steady state solution of the resultant kinetics is given by:

(S27) $[m_M] = \frac{1}{\delta}\cdot\left(\frac{\alpha_1[P_T^1]K_1^{-1}+\alpha_2[P_T^1][M]^n}{K_1^{-1}+[M]^n} + \frac{\alpha_3[P_T^2]K_2^{-1}K_3^{-1}+\alpha_4[P_T^2]K_3^{-1}[U]+\alpha_5[P_T^2][C][U]}{K_2^{-1}K_3^{-1}+K_3^{-1}[U]+[C][U]}\right)$

Furthermore, assuming that the time scale of protein production and degradation (Eq. (S18)) is much longer than the time scale of mRNA synthesis and degradation (Eq. (S15)), we substitute Eq. (S27) in Eq. (S18), resulting in :

(S28) $\frac{d[M]}{dt} = \frac{\beta}{\delta}\left(\frac{\alpha_1[P_T^1]K_1^{-1}+\alpha_2[P_T^1][M]^n}{K_1^{-1}+[M]^n} + \frac{\alpha_3[P_T^2]K_2^{-1}K_3^{-1}+\alpha_4[P_T^2]K_3^{-1}[U]+\alpha_5[P_T^2][C][U]}{K_2^{-1}K_3^{-1}+K_3^{-1}[U]+[C][U]}\right) - \mu_M[M]$

Denoting by $a_1^M = \frac{\beta}{\delta}\alpha_1[P_T^1]K_1^{-1}$, $a_2^M = \frac{\beta}{\delta}\alpha_2[P_T^1]$, $a_3^M = K_1^{-1}$, $a_1^{I_{\text{ext}}} = \frac{\beta}{\delta}\alpha_3[P_T^2]K_2^{-1}K_3^{-1}$, $a_2^{I_{\text{ext}}} = \frac{\beta}{\delta}\alpha_4[P_T^2]K_3^{-1}$, $a_3^{I_{\text{ext}}} = \frac{\beta}{\delta}\alpha_5[P_T^2]$, $a_4^{I_{\text{ext}}} = K_2^{-1}K_3^{-1}$, and $a_5^{I_{\text{ext}}} = K_3^{-1}$, Eq. (S28) is equivalent to Eq. (1).

2. Derivation of Eq. (3)

We assume that the expression of R is regulated by two promoters, denoted by P_R^1 and P_R^2. The promoter P_R^1 mediates the regulation of R by the US (UR pathway) such that its dynamics follow the TF-DNA binding kinetics:

(S29) $P_R^1 + U \underset{k_{-1}^R}{\overset{k_1^R}{\rightleftharpoons}} P_R^1 U$

The corresponding kinetics are:

(S30) $\frac{d[P_R^1 U]}{dt} = k_1^R [P_R^1][U] - k_{-1}^R [P_R^1 U]$

with the conservation equation

(S31) $[P_T^{R,1}] = [P_R^1] + [P_R^1 U]$

The promoter P_R^2 is regulated by proteins *C* and *M* (CR pathway):

(S32) $P_R^2 + C \underset{k_{-2}^R}{\overset{k_2^R}{\rightleftharpoons}} P_R^2 C$

(S33) $P_R^2 + nM \underset{k_{-3}^R}{\overset{k_3^R}{\rightleftharpoons}} P_R^2 M_n$

(S34) $P_R^2 C + nM \underset{k_{-4}^R}{\overset{k_4^R}{\rightleftharpoons}} P_R^2 C M_n$

The two promoters P_R^1 and P_R^2 regulate the synthesis of *R*'s mRNA, m_R, which is translated into protein *R*. The synthesis and degradation equations of m_R are given by:

(S35) $P_R^1 \overset{\alpha_1^R}{\rightarrow} m_R$

(S36) $P_R^1 U \overset{\alpha_2^R}{\rightarrow} m_R$

(S37) $P_R^2 \overset{\alpha_3^R}{\rightarrow} m_R$

(S38)$P_R^2 C \overset{\alpha_4^R}{\rightarrow} m_R$

(S39) $P_R^2 M_n \overset{\alpha_5^R}{\rightarrow} m_R$

(S40) $P_R^2 C M_n \overset{\alpha_6^R}{\rightarrow} m_R$

(S41) $m_R \overset{\delta_R}{\rightarrow} \phi$

The production and degradation of protein *R* are simply given by:

(S42) $m_R \overset{\beta_R}{\rightarrow} R$

(S43) $R \overset{\mu_R}{\rightarrow} \phi$

As in the derivation of Eq. (1), we assume that $[U] \gg [P_R^1]$ and $[C], [M] \gg [P_R^2]$ and therefore:

(S44) $[U_T] = [U] + [P_R^1 U] \approx [U]$

(S45) $[M_T] = [M] + n \cdot [P_R^2 M_n] + n \cdot [P_R^2 C M_n] \approx [M]$

(S46) $[C_T] = [C] + [P_R^2 C] + [P_R^2 C M_n] \approx [C]$

Moreover, we assume that (1) the time-scales of the TF-DNA bindings (Eqs. (S29) and (S32)-(S34)) are much shorter than the time scale of mRNA synthesis and degradation (Eqs. (S35)-(S41)) and (2) the time scale of protein production and degradation (Eq. (S42)- (S43)) is much longer than the preceding time scales.

With these assumptions we substitute the steady state solutions of the TF-DNA bindings in the mRNA synthesis equations and the state solutions of the mRNA synthesis in the protein production and degradation equation, yielding

(S47)

$$\frac{dR}{dt} = \frac{\beta_R}{\delta_R} \cdot \left(\frac{\alpha_1^R [P_T^{R,1}] K_{1,R}^{-1} + \alpha_2^R [P_T^{R,1}][U]}{K_{1,R}^{-1} + [U]} + \frac{\alpha_3^R [P_T^{R,2}] K_{2,R}^{-1} K_{4,R}^{-1} + \alpha_4^R [P_T^{R,2}] K_{4,R}^{-1}[C] + \alpha_5^R [P_T^{R,2}] K_{2,R}^{-1} K_{3,R} K_{4,R}^{-1} [M]^n + \alpha_6^R [P_T^{R,2}][C][M]^n}{K_{2,R}^{-1} K_{4,R}^{-1} + K_{4,R}^{-1}[C] + K_{2,R}^{-1} K_{3,R} K_{4,R}^{-1}[M]^n + [C][M]^n} \right) - \mu_R \cdot [R]$$

Denoting by $a_1^U = \frac{\beta_R}{\delta_R} \alpha_1^R [P_T^{R,1}] K_{1,R}^{-1}$, $a_2^U = \frac{\beta_R}{\delta_R} \alpha_2^R [P_T^{R,1}]$, $a_3^U = K_{1,R}^{-1}$, $a_1^{CM} = \frac{\beta_R}{\delta_R} \alpha_3^R [P_T^{R,2}] K_{2,R}^{-1} K_{4,R}^{-1}$, $a_2^{CM} = \frac{\beta_R}{\delta_R} \alpha_4^R [P_T^{R,2}] K_{4,R}^{-1}$, $a_3^{CM} = \frac{\beta_R}{\delta_R} \alpha_5^R [P_T^{R,2}] K_{2,R}^{-1} K_{3,R} K_{4,R}^{-1}$, $a_4^{CM} = \frac{\beta_R}{\delta_R} \alpha_6^R [P_T^{R,2}]$, $a_5^{CM} = K_{2,R}^{-1} K_{4,R}^{-1}$, $a_6^{CM} = K_{4,R}^{-1}$ and $a_7^{CM} = K_{2,R}^{-1} K_{3,R} K_{4,R}^{-1}$, Eq. (S47) is equivalent to Eq. (3).

3. Derivation of Eq. (5)

For the derivation of generalized CR pathway in Eq. (5) (the last term in the equation) we assume for simplicity N independent promoters that regulate R. Each of them acts in a similar manner to the CR term in the single pathway model (G_2). Denoting by $a_1^U = \frac{\beta_R}{\delta_R} \alpha_1^R [P_T^{R,1}] K_{1,R}^{-1}$, $a_2^U = \frac{\beta_R}{\delta_R} \alpha_2^R [P_T^{R,1}]$, $a_3^U = K_{1,R}^{-1}$, $a_1^{CM} = N \cdot \frac{\beta_R}{\delta_R} \alpha_3^R [P_T^{R,2}] K_{2,R}^{-1} K_{4,R}^{-1}$, $a_2^{CM} = N \cdot \frac{\beta_R}{\delta_R} \alpha_4^R [P_T^{R,2}] K_{4,R}^{-1}$, $a_3^{CM} = N \cdot \frac{\beta_R}{\delta_R} \alpha_5^R [P_T^{R,2}] K_{2,R}^{-1} K_{3,R} K_{4,R}^{-1}$, $a_4^{CM} = N \cdot \frac{\beta_R}{\delta_R} \alpha_6^R [P_T^{R,2}]$, $a_5^{CM} = K_{2,R}^{-1} K_{4,R}^{-1}$, $a_6^{CM} = K_{4,R}^{-1}$ and $a_7^{CM} = K_{2,R}^{-1} K_{3,R} K_{4,R}^{-1}$–, the generalized form of Eq. (S47), with multiple promoters, is equivalent to Eq. (6).

It should be noted that Eq. (5) is also the deterministic approximation of other processes that do not entail the assumption of different promoters. However, in this case there are further assumptions on the values of the kinetic parameters. Moreover, it should be noted that the capacity calculations remain unchanged as long as the expression of R is a monotonic increasing function of the number of bounded complexes $C_i - M_i$.

4. Derivation of Eqs. (8-9)

In order to derive Eqs. (8-9), we first consider the four possible contribution of each pathway:

(S48)

$$R_{00} = \frac{a_1^{CM}+a_2^{CM}[C^{low}]+a_3^{CM}[M^{low}]^n+a_4^{CM}[C^{low}][M^{low}]^n}{a_5^{CM}+a_6^{CM}[C^{low}]+a_7^{CM}[M^{low}]^n+[C^{low}][M^{low}]^n}$$

$$R_{01} = \frac{a_1^{CM}+a_2^{CM}[C^{low}]+a_3^{CM}[M^{high}]^n+a_4^{CM}[C^{low}][M^{high}]^n}{a_5^{CM}+a_6^{CM}[C^{low}]+a_7^{CM}[M^{high}]^n+[C^{low}][M^{high}]^n}$$

$$R_{10} = \frac{a_1^{CM}+a_2^{CM}[C^{high}]+a_3^{CM}[M^{low}]^n+a_4^{CM}[C^{high}][M^{low}]^n}{a_5^{CM}+a_6^{CM}[C^{high}]+a_7^{CM}[M^{low}]^n+[C^{high}][M^{low}]^n}$$

$$R_{11} = \frac{a_1^{CM}+a_2^{CM}[C^{high}]+a_3^{CM}[M^{high}]^n+a_4^{CM}[C^{high}][M^{high}]^n}{a_5^{CM}+a_6^{CM}[C^{high}]+a_7^{CM}[M^{high}]^n+[C^{high}][M^{high}]^n}$$

Thus, $G_2([C_i],[M_i])$ can be written as:

(S49) $G_2([C_i],[M_i]) = \frac{a_1^{CM}+a_2^{CM}\cdot[C_i]+a_3^{CM}\cdot[M_i]^n+a_4^{CM}\cdot[C_i][M_i]^n}{a_5^{CM}+a_6^{CM}\cdot[C_i]+a_7^{CM}\cdot[M_i]^n+[C_i]\cdot[M_i]^n} = (1-c_i)\cdot(1-m_i)\cdot R_{00} + (1-c_i)\cdot m_i\cdot R_{01} + c_i\cdot(1-m_i)\cdot R_{10} + +c_i\cdot m_i\cdot R_{11}$

Rewriting Eq. (S49) yields:

(S50) $G_2([C_i],[M_i]) = R_{00} - \frac{(R_{10}-R_{00})(R_{01}-R_{00})}{(R_{11}+R_{00}-R_{10}-R_{01})} + (R_{11}+R_{00}-R_{10}-R_{01})\cdot\left(c_i m_i - \frac{-(R_{10}-R_{00})}{(R_{11}+R_{00}-R_{10}-R_{01})}c_i - \frac{-(R_{01}-R_{00})}{(R_{11}+R_{00}-R_{10}-R_{01})}m_i + \frac{(R_{10}-R_{00})(R_{01}-R_{00})}{(R_{11}+R_{00}-R_{10}-R_{01})^2}\right)$

yielding Eqs. (8-9).
